# Virtual Reality for Pre-Procedural Planning of Interventional Pain Procedures: A Real-World Application Case Series

**DOI:** 10.3390/jcm14093019

**Published:** 2025-04-27

**Authors:** Ingharan J. Siddarthan, Cary Huang, Parhesh Kumar, John E. Rubin, Robert S. White, Neel Mehta, Rohan Jotwani

**Affiliations:** 1 Department of Anesthesiology, NewYork-Presbyterian Hospital and Weill Cornell Medicine, New York, NY 10065, USA; ijs9006@nyp.org (I.J.S.); ndu9002@nyp.org (C.H.); 2 Department of Anesthesiology, Weill Cornell Medicine, New York, NY 10065, USA; pak4011@med.cornell.edu (P.K.); jer9173@med.cornell.edu (J.E.R.); rsw9006@med.cornell.edu (R.S.W.); nem9015@med.cornell.edu (N.M.)

**Keywords:** augmented reality (AR), custom patient models, digital imaging and communications in medicine (DICOM), extended reality (XR), mixed reality (MR), pain medicine, surgery, three-dimensional (3D)

## Abstract

**Background/Objectives**: Virtual reality (VR), a component of extended reality (XR), has shown promise in pre-procedural planning by providing immersive, patient-specific simulations. In pain management, where precise anatomical understanding is critical for interventions such as peripheral nerve stimulation (PNS), nerve blocks, and intrathecal pump placement, the application of VR remains underexplored. This case series examines the role of VR in enhancing pre-procedural planning for complex chronic pain interventions. **Methods**: From August 2022 to December 2024, six patients with anatomically challenging conditions underwent VR-assisted pre-procedural planning at Weill Cornell Medical Center. Patient-specific 3D models were created using the manual or automatic segmentation of imaging data and reviewed in VR to optimize procedural strategies by the surgeons performing the case. Procedures were then performed using conventional fluoroscopic or ultrasound guidance. **Results**: In all cases, VR facilitated the improved visualization of complex anatomies and informed optimal procedural trajectories. In patients with a complex cancer anatomy, previous surgical changes, or hardware, VR enabled precise PNS lead or needle placement, resulting in significant pain reductions postoperatively. In certain cases where previous interventional pain procedures had failed, VR allowed for a “second opinion” to develop an alternative approach with improved outcomes. Finally, in one case, VR served to potentially prevent patient harm by providing insight to the proceduralists regarding an alternative approach. Across the series, VR enhanced the spatial awareness, procedural accuracy, and confidence in navigating challenging anatomical scenarios. **Conclusions**: This case series demonstrates the utility of VR in pre-procedural planning for chronic pain interventions. By enabling detailed anatomical visualization and trajectory optimization, VR has the potential to improve outcomes in complex cases. Further studies are needed to evaluate its broader clinical applications and cost-effectiveness in pain management.

## 1. Introduction

Advancements in immersive technologies—specifically virtual reality (VR), augmented reality (AR), and mixed reality (MR), collectively known as extended reality (XR)—have significantly expanded their application in pre-procedural planning across various medical specialties. VR provides fully immersive simulations, allowing physicians to practice procedures and understand complex anatomies within a risk-free environment [1]. Recent reviews have highlighted the effectiveness of VR in preoperative planning, particularly in visualizing patient-specific anatomies and aiding complex surgical cases [1,2]. In contrast, AR overlays digital information onto the real world, assisting in the real-time visualization of anatomical structures during planning [3]. MR combines virtual and real elements, enabling the interactive manipulation of patient-specific models for collaborative planning. Together, these technologies enhance physicians’ abilities to plan and execute procedures with greater accuracy, potentially improving patient outcomes [4].

Currently, VR technology is being utilized by multiple specialties, including cardiothoracic and neurosurgery, as a tool for pre-procedural planning in patients with an atypical anatomy [1,2,5]. For instance, in cardiac surgery, VR offers immersive three-dimensional imaging that assists in the planning of minimally invasive procedures [5]. The application of virtual reality for the pre-procedural planning of interventional pain procedures, however, is still in its infancy. Our team recently highlighted the application of VR in planning for a thoracic nerve root block [3], illustrating the potential for this technology within the field of pain medicine.

In the field of pain management, procedures such as peripheral nerve blocks, peripheral nerve stimulation (PNS), neuraxial interventions, and intrathecal pump placement are critical in treating chronic pain and necessitate a precise anatomical understanding and meticulous pre-procedural planning. These procedures become even more complex or risky in patients with anatomically challenging conditions, such as mass-occupying lesions from cancer or the presence of surgical hardware. Peripheral nerve blocks, or single-shot injections of peripheral nerves, can be straightforward procedures when the usual anatomy is present, but these otherwise safe procedures can become difficult when mass-occupying lesions are present or the usual anatomic landmarks are absent. PNS involves placing small electrodes near peripheral nerves to deliver electrical impulses that modulate pain signals, effectively reducing chronic pain in targeted areas [6]. Precise lead deployment is essential to selectively target the nerve of interest, a challenging task in complex anatomies. Neuraxial interventions, such as epidural injections or spinal cord stimulation, aim to alleviate pain by delivering medication or electrical stimulation directly to central nervous system pathways. Intrathecal pump placement entails surgically implanting a programmable pump and catheter system that delivers pain medication directly into the intrathecal space surrounding the spinal cord, allowing for precise dosing and reduced systemic side effects in cases of severe, intractable pain [7].

Conventional cross-sectional imaging plays an integral role in pre-procedural planning, but the evaluation of the most complex cases is limited by the cuts offered in just two dimensions (axial, coronal, sagittal). Given the complexity of these interventions, VR technology offers significant benefits by enhancing the planning process through immersive, patient-specific simulations [4]. VR improves spatial awareness and procedural accuracy, ultimately optimizing outcomes and minimizing risks in pain management procedures [8]. Additionally, VR provides an interactive and collaborative environment for clinicians to study, manipulate, and annotate the patient-specific anatomy in three dimensions [2]. The increasing commercial availability of head-mounted displays (HMDs), with enhanced rendering power and clarity, further improves the accessibility of these technologies. With operation on portable, low-cost, commercially available hardware, VR technology is now more feasible for widespread clinical use.

In this paper, we present a case series of six patients for whom VR was integral in planning and executing chronic pain interventions, including peripheral nerve stimulation, nerve blocks, and intrathecal pump placement. Our aim is to highlight how VR technology enhances preprocedural planning, discuss its benefits and limitations, and explore future directions for its application in pain management procedures.

## 2. Materials and Methods

### 2.1. Patient Selection

From August 2022 to December 2024, a total of six patients underwent pre-procedural planning in the context of interventional pain medicine at Weill Cornell Medical Center/New York Presbyterian Hospital with the use of XR technology. For all six patients, a case-specific 3D model was created by either the manual or automatic segmentation of the relevant anatomy described below. The six patients were chosen based on the proceduralists’ (authors R.J. and J.R.) discretion for cases where it was believed that three-dimensional (3D) modeling would be impactful and beneficial in procedural planning. This decision was based on affirmative answers to the following questions:

- Does the intervention require pre-procedural imaging and planning?
- Does the case present sufficient variation from the standard intervention such that a new methodology of pre-procedural planning might be beneficial?
- Does the case present any of the following variables: anatomical changes due to previous surgery or past or present malignancy, a previously failed attempt at the intervention being considered, or a potential risk where modeling the 3D trajectory may decrease harm intra-procedurally?

The creation of a digital twin was only considered if both authors agreed on the responses to all three questions. For each case, patient consent was obtained prior to any model creation. The team obtained an IRB exclusion on the basis that (1) the cases presented amounted to less than ten; (2) the investigation here did not constitute a “systematic investigation”; and (3) the results described did not represent “generalizable knowledge”.

### 2.2. Image Reconstruction

Three-dimensional (3D) models for each patient were generated in VR using either a manual segmentation approach via “Elucis v 1.6.1 © Realize Medical, Ottawa, ON, Canada” (Cases 1, 4) or a volumetric-based, automatic segmentation approach using the “Medical Imaging XR v 1.5.0 © MEDICALHOLODECK Inc., Zurich, Switzerland” software (Cases 2, 3, 5, 6). Relevant computed tomography (CT) imaging studies for each case were first imported in anonymized Digital Imaging and Communications in Medicine (DICOM) format. For the majority of cases, high-resolution CT scans were acquired using a 64-slice CT scanner (Siemens Medical Solutions, Malvern, PA, USA), with a slice thickness of 0.5 mm and a reconstruction interval of 0.3 mm. For one case, MRI images were utilized from an outside hospital record; the exact specifications and machine used were unknown. Then, the DICOM files were uploaded into a HIPAA-compliant cloud server from the respective software platform. Once the anonymized files were uploaded to the software, head-mounted displays (HMDs; specifically, the Meta Quest 2 or 3 © Meta, Inc., Menlo Park, CA, USA) were utilized to reconstruct, modify, or interpret any 3D models. Personalized computer VR was required to perform tasks in Elucis using a tethered connection to a Lenovo Thinkpad © P1 Gen5 Laptop (Morrisville, NC, USA) with an Intel© Core i7-12700H processor (Santa Clara, CA, USA) and a NVIDIA© RTX A1000 (Santa Clara, CA, USA) graphics card. Standalone VR was utilized.

In the volumetric based (Medical Holodeck) software, the segmentation of the imaging data was automatically performed, translating 2D radiographic windows into 3D representations of tissue, which were differentiated by varying color and saturation in the XR space. The platform’s artificial intelligence (AI)-powered automatic segmentation algorithm (open-source totalsegementor, University of Basel, Switzerland) was applied to identify key anatomical structures. This platform utilizes a nnU-Net framework and was trained on a diverse dataset of CT scans from various scanners, institutions, and protocols, enabling accurate segmentation across a wide range of clinical scenarios based on previous descriptions [9]. Manual corrections were performed by experienced pain interventionalists to ensure accuracy, particularly in regions of surgical interest. The resulting data could then be viewed in VR as a volumetric model displaying the entire image in 3D. The model could be manipulated in all dimensions. A “transect” feature provided the dynamic examination of individual cross-sections of the image, which were selected via a pass-through motion and functioned in any direction. Additionally, the models were able to be altered via global adjustments to radiographic windowing, adding labels and markers to the image, and scaled measurements of structures within the model.

The manual segmentation (Elucis) software allowed for manual segmentation to generate customized models. When initially imported, the two-dimensional (2D) imaging data were automatically translated into a grayscale 3D holographic model with adjustable radiographic windowing. Then, the model could be manually rotated and passed through a 2D “drawing” plane in any direction by the operator. The cross-section selected in the drawing plane could then be highlighted with selected colors and textures to delineate structures and tissue types of interest by limited structures within certain Hounsfield unit windows. The manual reconstruction of the 3D model by the operator was more time- and labor-intensive than the AI-based reconstruction provided by the volumetric reconstruction software. The advantage of this approach, however, was that customizable 3D models of the patient’s anatomy could be generated with specific foci on contrasting areas of interest (e.g., differentiating surgical hardware from bone).

### 2.3. Image Review and Pre-Procedural Planning

After creating 3D models for each patient, the proceduralists then reviewed the models in VR to plan an interventional approach. The procedures were then conducted under traditional fluoroscopy or ultrasound guidance using conventional practices. Thus, beyond the pre-procedural planning phase, no aspect of the operative practice was altered. Augmented reality was not utilized, and the models were not available for review within procedural or operative environments. No models were created post-procedurally.

## 3. Results

### 3.1. Case 1

A 67-year-old male presented with a 5-year history of neck pain radiating to the left shoulder and left occiput. He had a complex surgical history, including three prior cervical spine surgeries: C3–C7 fusion in 2015, revision/extension to C2–C7 in 2017, and further extension of fusion to encompass C2-T2 in 2018. He reported primarily axial neck pain, which had worsened since his surgeries. The exam showed a fixed cervical kyphosis, hypertrophied and tender muscles left-lateral to his cervical incision, and tenderness along the left trapezius and posterior neck. Imaging showed a posterior hardware construct consisting of a left lateral mass screw and right-sided compression hook at C2, bilateral lateral mass screws at C4–C6, a right lateral mass screw at C7, and bilateral pedicle screws at T1 and T2. He was fused with a cervical kyphosis, with grade 1 anterior spondylolisthesis of C3 over C4 and osteophytes throughout the cervical spine.

**Figure 1:**
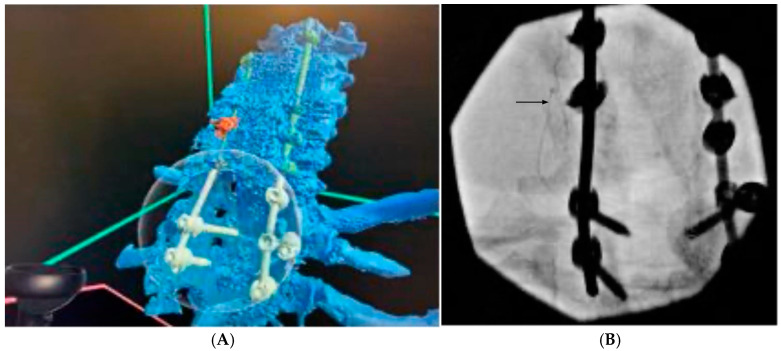
Patient 1, who underwent cervical medial branch PNS for chronic axial postoperative neck pain. (**A**) Segmental reconstruction of patient imaging in 3D demonstrated extensive hardware and laminectomies. The model revealed that placing a lead at C5/6, with the lateral mass screws taking up space nearby, would be challenging to deploy and of high risk for migration. Therefore, the decision was made to enter closer to C7, where hardware was absent, and aim the lead towards the C5/6 facet joint. (**B**) Successful lead deployment under fluoroscopy. Arrow indicates PNS lead.

He was diagnosed with postoperative neck pain with an average intensity of 8/10 on the Numerical Rating Scale (NRS). Given his primarily axial neck pain and the localization of pain to the left C5/6/7 facet joints, an initial medial branch block was performed, with a good result. PNS was selected for treatment given the success of the peripheral block and the perilous anatomy for central neuromodulation. PNS leads require precise placement near target nerves and adequate tissue to hold them in place—both of which were challenging to ascertain from the cross-sectional imaging alone. VR was therefore used in this patient to closely examine his spine and find the most likely path for successful lead deployment. Manual segmentation was utilized to reconstruct a three-dimensional model of the patient’s spine from imaging, with special attention to the location of pedicle screws (Figure 1A). Manual segmentation (Elucis©) was utilized here to allow the operator to distinguish between objects of similar radiodensities—specifically, hardware and bone. Exploring the model in 3D allowed the identification of a safe path for needle entry closer to C7, with a parasagittal oblique trajectory to ultimately deploy the lead at the C5/6 facet joint. Lead deployment was successful (Figure 1B). Intraoperatively and immediately postoperatively, the patient reported the near-total resolution of his pain, although the leads were ultimately discontinued due to migration one month postoperatively.

### 3.2. Case 2

**Figure 2:**
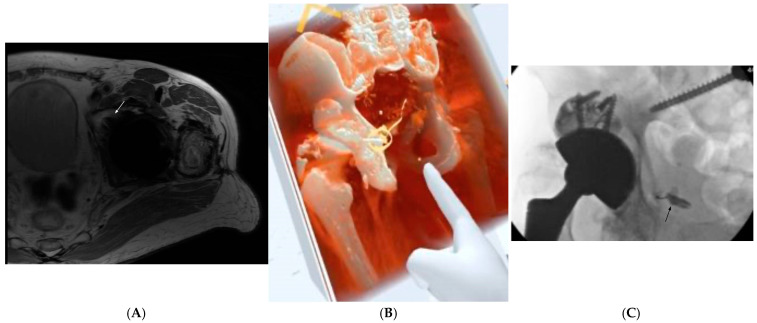
Patient 2, who had persistent pudendal neuralgia following a left total hip replacement and subsequent removal of an acetabular screw causing contact with the pudendal neurovascular bundle. (**A**) MR of the left hip showed that the acetabular screw (white arrow) was slightly deep and impinging on Alcock’s canal near the ischial spine. (**B**) Volumetric reconstruction of patient CT imaging following screw removal. Since the primary anatomic landmark for fluoroscopy-guided injection was removed (ischial spine), VR was utilized to obtain measurements and distances to reach Alcock’s canal in order to perform a pudendal block. (**C**) Fluoroscopy image of successful contrast and medication delivery in the pudendal canal. Arrow indicates block site.

A 72-year-old male with a history of multiple lumbar spine and hip surgeries presented with two years of severe 10/10 anorectal pain that worsened when sitting, with no pain on direct examination of the rectum. His surgical history included three lumbar surgeries resulting in L3-S1 fusion, a total hip replacement, and a hip replacement revision. MRI of the left hip revealed an acetabular screw compressing the pudendal neurovascular bundle at Alcock’s canal, where the nerve travels anteriorly towards the perineum (Figure 2A). He then underwent an operation to remove this screw, along with the ischial spine. Unfortunately, he had persistent pain despite the removal of the offending hardware and was diagnosed with chronic pudendal neuralgia. He underwent epidural steroid injections, a ganglion impar block, and an attempted pudendal block without relief.

In addition to the presence of hardware, the absence of the typical landmark of a fluoroscopic pudendal nerve block (ischial spine) contributed to the complexity of this case. The patient’s postoperative CT was converted to a 3D model using volumetric reconstruction (Figure 2B). The primary issue to address was the optimal angle of approach and distance to traverse to enter Alcock’s canal and perform a successful block. The distances from the skin to the block site were measured at multiple areas and angles. Ultimately, the procedure was performed by walking off the ischium between the greater and lesser sciatic foramina, corresponding to the screw path, until the distance approximated the estimates obtained in VR. Contrast injection confirmed the correct needle placement, and the block medication was administered (Figure 2C). Although the patient reported only 10% pain relief from the initial 10/10 pain, he noted significant functional improvements, including the ability to sit to drive and ride public transportation for the first time in years, without the exacerbation of pain.

### 3.3. Case 3

**Figure 3:**
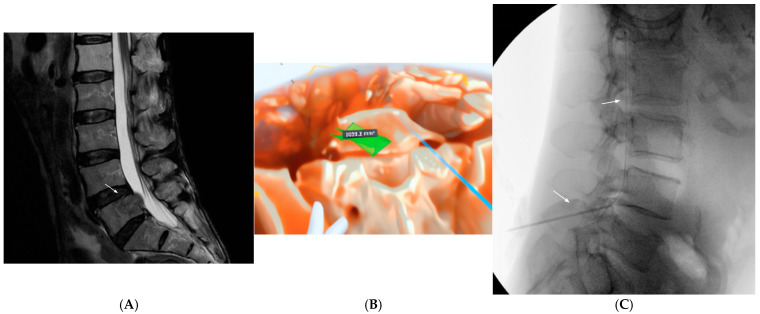
Patient 3, who did not tolerate the adverse effects of opioid therapy required for cancer pain and underwent intrathecal pump placement. (**A**) T2-weighted sagittal MR image demonstrating lytic lesion at L5 (white arrow). (**B**) Volumetric reconstruction of patient imaging, with polygons used to demarcate the tumor and planning of the path of entry. (**C**) Successful intrathecal pump placement using an L3/4 right paramedian approach. White arrows delineate the path of the intrathecal catheter.

A 67-year-old male with an advanced metastatic gastric adenocarcinoma was hospitalized with pain due to bony metastasis to the L5 vertebral body and left pedicle causing the compression of the left L5 nerve root and severe left leg pain. He received radiation to the area and was not a surgical candidate. He was on high-dose opioids for pain control but had recurrent hospital admissions due to the adverse effects of opioid therapy and pain crises. MR of the spine revealed metastatic lesions causing bony destruction at the T9 and L5 vertebral bodies and L5 left pedicle and nerve root invasion (Figure 3A). The pain management service was consulted to determine whether an intrathecal drug delivery system could be placed despite the presence of metastatic cancer of the spine.

Here, the challenge posed was placing the pump in a location that could deliver the intrathecal opioid to most of the spine, while entering in a safe location to avoid the metastases. Volumetric reconstruction was used to model this patient, with several goals: to obtain the 3D dimensions of the L5 lesion, to assess the degree of invasion into the L4/5 posterior complex, and to measure the distance from the skin to the intrathecal space at several angles. The tumor was identified by noting irregularities in the reconstructed spine and demarcating them with green polygon tracings (Figure 3B). The ruler function was then used to plan different paths and select the level of entry. Compared with measurements in conventional imaging, measurements in 3D allow for distances to be obtained in three axes, mimicking the true needle trajectory. Ultimately, a right paramedian approach at L3/4 was planned. The lesions were significantly less clear than under fluoroscopy; therefore, the careful analysis of the model preoperatively also reassured the team that the needle was in a safe location at all times. The operation was uncomplicated (Figure 3C). The patient reported subjective improvements in cancer pain. Of note, following intrathecal drug delivery initiation, the patient did not have any further admissions to this institution for oversedation or pain crises.

### 3.4. Case 4

**Figure 4:**
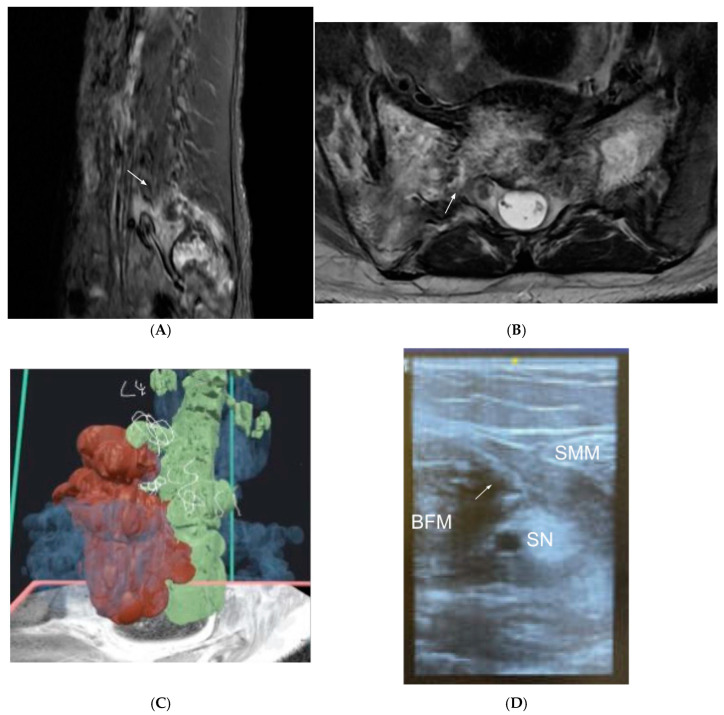
Patient 4, who developed significant radicular pain in the sciatic nerve distribution from a large, destructive lymphoma, causing spinal and foraminal stenosis. (**A**,**B**) Initial imaging prior to chemotherapy showing a sacral mass with epidural extension and significant right > left L5/S1 foraminal stenosis. White arrows indicate the mass. (**C**) Manual segmentation was utilized to generate a 3D model of the patient’s spine MR. Grayscale was manually converted to color by our team, with blue representing bone, green representing neural structures, and red demarcating the tumor. (**D**) Ultrasound-guided sciatic PNS lead deployment near the popliteal fossa. BFM = biceps femoris muscle, SN = sciatic nerve, SMM = semimembranosus muscle, white arrow indicates PNS lead.

A 31-year-old transmasculine patient with diffuse large B-cell lymphoma presented with sciatic nerve pain due to the involvement of the sacrum, lumbar spine, and spinal cord. He reported primarily right-sided radicular pain along the low lumbar and sacral nerve root distributions. At the initial consultation, he required high-dose opioid therapy and multiple neuropathic pain medications to tolerate the pain. Initial MR imaging revealed a large, destructive mass with epidural involvement causing spinal stenosis at L4/5 and L5/S1 and primarily right foraminal stenosis at L5/S1 (Figure 4A,B). He underwent systemic chemotherapy, which significantly reduced the mass size but did not improve his pain syndrome. Due to his young age and robust response to chemotherapy, the patient and pain team opted to trial neuromodulation therapies to bridge an opioid wean and avoid intrathecal drug delivery.

Here, the problem addressed with VR was to more closely examine the spine and determine which, if any, neuromodulation options between spinal cord stimulation, dorsal root ganglion stimulation, and PNS were viable for this patient. To examine the structures in detail, manual segmentation was utilized to build the patient’s spine in 3D and clearly demarcate tumor involvement in the epidural space, neuroforamina, and bony structures (Figure 4C). A grayscale 3D model was automatically generated, and then the axial MRI was “passed through” the model, with color utilized to delineate the different structures at each cut. Here, green was used to represent nerve structures, blue to represent bone, and red to represent the tumor. Areas of contact were more closely assessed in multiple dimensions. Close review confirmed that neither an epidural nor a dorsal root ganglion lead placement would safely avoid the patient’s tumor. Because the patient’s pain was primarily in the lower leg and foot, a popliteal sciatic PNS was instead considered to offer some pain relief (Figure 4D).

The procedure was performed successfully; the patient achieved 40% pain relief (from initial 10/10 pain) at 3 months and was eventually transitioned from full opioid agonist therapy to partial agonist therapy (buprenorphine). This case therefore demonstrates the value of VR and 3D modeling in making a decision to abort a neuraxial procedure in favor of a safer peripheral procedure. The discussion of this modeling with the patient and the subsequent shared decision-making built trust between him and the pain team, which likely aided in subsequent opioid weaning efforts.

### 3.5. Case 5

A 74-year-old female presented with a year of ongoing, constant right-sided pelvic pain after the complex repair of a right rectus abdominis tendon tear, revision of the prior right inguinal hernia repair, and neurectomy of the iliohypogastric nerve. Additionally, she developed a large abdominal seroma postoperatively, with the entrapment of the genitofemoral nerve. She also notably had a T2-S1 posterior instrumented fusion performed many years prior for severe scoliosis. She failed more conservative management, including physical therapy and medical management, but had responded well to ilioinguinal and iliohypogastric nerve blocks. As her history of prior T1-S1 spinal fusion precluded the placement of a spinal cord stimulator, she instead underwent the implantation of PNS leads.

**Figure 5:**
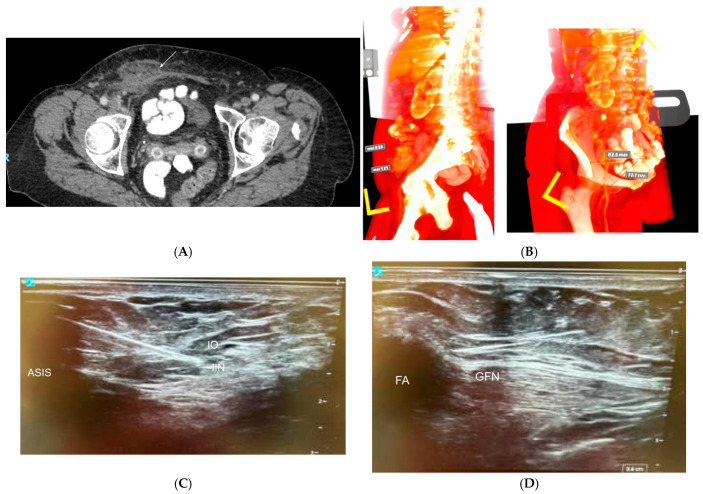
Patient 5, who underwent 2-lead PNS implantation for chronic abdominal wall pain. (**A**) CT scan demonstrating seroma adjacent to prior right inguinal hernia repair (white arrow). Neurectomy of iliohypogastric nerve also occurred during this operation. (**B**) Volumetric reconstruction of patient CT abdomen, utilized to plan the best path for lead deployment prior to the operation. (**C**) Ultrasound-guided ilioinguinal/iliohypogastric nerve PNS deployment. ASIS = anterior superior iliac spine, IIN = ilioinguinal nerve, IO = internal oblique muscle. (**D**) Ultrasound-guided genitofemoral nerve PNS deployment. GFN = genitofemoral nerve, FA = femoral artery.

However, given her adjacent seroma, prior surgical dissections, and the extensive atrophy of the abdominal wall muscles, lead placement was anticipated to be extremely technically challenging, as PNS leads require centimeters of tunneling in soft tissue to remain in place. A model of her CT abdomen was created using volumetric reconstruction, with the specific goal of identifying safe locations for lead tunneling and placement despite her many prior abdominal wall surgeries (Figure 5B). Two lead trajectories were modeled: the first adjacent to the right ilioinguinal and iliohypogastric nerves, which typically run close to the anterior superior iliac spine, and the second near the genital branch of the right genitofemoral nerve, adjacent to the femoral artery. In both cases, medial to lateral approaches were modeled as these allowed for between 6 and 8 cm of lead space between entry and the potential target without requiring depth that went beyond the abdominal wall. The patient underwent the implantation of the leads operatively (Figure 5C,D), with the planned trajectories utilized to provide safe placement without the risk of peritoneal puncture. She experienced pain relief from an average of NRS 7/10 abdominal pain preoperatively to an average of 4/10 postoperatively. Her leads were ultimately discontinued due to an unanticipated allergic reaction to the metal within the lead.

### 3.6. Case 6

**Figure 6:**
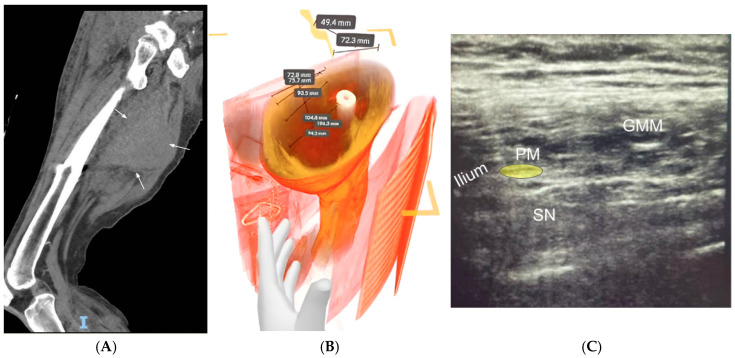
Patient 6, who underwent an ultrasound-guided sciatic nerve block for sciatic neuralgia caused by a large sarcoma involving the adductor musculature. (**A**) CT scan demonstrated the involvement of most of the pathway of the sciatic nerve by the tumor (white arrows). (**B**) Virtual three-dimensional reconstruction of the CT scan. The mass was measured in multiple dimensions, taking special note of the cephalad extent of the tumor. A modified subgluteal sciatic approach, closer to the greater sciatic foramen, was planned. (**C**) Ultrasound-guided block of the sciatic nerve deep to piriformis, safely above the superior margin of the tumor. GMM = gluteus maximus muscle, PM = piriformis muscle, SN = sciatic nerve.

A 67-year old male with a recently diagnosed, untreated, right thigh spindle cell sarcoma presented with new-onset shortness of breath, upper-extremity edema, and facial plethora while awaiting treatment for his cancer. He was found to have a large metastasis involving the right atrium and superior vena cava and was admitted to our cardiothoracic intensive care unit for the management of superior vena cava syndrome. He had significant radicular pain from the primary tumor in his leg, primarily in the sciatic distribution, which prevented meaningful participation in physical therapy and resulted in significant deconditioning. Intravenous medication therapy for pain was limited due to his respiratory symptoms. CT imaging of the thigh revealed a 11.3 × 10.5 × 16.4 cm heterogenous mass located within the common adductor musculature, with the likely compression of the sciatic nerve (Figure 6A).

A sciatic nerve block was considered for pain relief, but a safe location for the block was difficult to identify due to the size (and location) of the mass within the common adductor musculature. Specifically, it was unclear where the sciatic nerve could be safely blocked given the large size of the tumor. Volumetric reconstruction was used to assess the size of the mass and cephalocaudal involvement (Figure 6B). The problem addressed by VR in this case was whether a sacral sciatic or similar block could be safely performed, as it appeared that even a transgluteal or subgluteal approach would have been too close to the tumor. Model review revealed that the mass primarily occupied the upper and mid-thigh, but the gluteal region was spared.

The sciatic nerve was blocked distal to the greater sciatic foramen, where it courses deep to the piriformis muscle, with local anesthetic and methylprednisolone (Figure 6C). In the days following the block, the patient reported significant pain relief, but more notable was his significantly improved participation in physical therapy. Within several days of the block, he was able to stand for the first time since admission to the hospital and spend much more time out of bed in a chair. Ultimately, he was able to begin chemotherapy for his metastatic disease.

## 4. Discussion

The cases that we highlight here demonstrate that VR can offer an immersive and interactive environment for the careful analysis of complex anatomies and for the planning of potential interventions. Case 1 demonstrated the value of VR in planning a path to ensure the maximal success rate for PNS deployment around complex surgical hardware. Case 2 allowed for the planning of a block in the absence of the usual fluoroscopic landmarks. Case 3 affirmed the safety of intrathecal pump placement in a patient with metastatic cancer to the neuraxis and allowed for the prediction of distances to the intrathecal space at safe locations to save time intraoperatively. Case 5 was similar to case 1 in that VR allowed the selection of the optimal path of PNS lead placement to minimize the risk of lead migration and injury to a surgically altered abdominal wall. Case 6 allowed the selection of the optimal location to safely and effectively block a nerve to allow a patient with cancer to achieved an improved functional status and rehabilitate prior to beginning systemic chemotherapy. Case 4 was especially insightful, as it taught the team how 3D modeling in VR can affirm the decision not to perform a procedure and instead select safer options for a patient. Many patients appreciate the review of their own imaging in the clinic; a review of their specific imaging in 3D and an understanding of the care placed in planning their procedures served to build trust between the patient and the care team. Future studies may consider examining whether incorporating XR patient education with the patient’s specific digital twin may improve their understanding of the procedure, therefore improving the consent process and the patient’s engagement with treatment.

The two VR software implementations that we used provided different approaches to creating a 3D model from 2D imaging data. Medical Holodeck© allowed for easily accessible volumetric reconstruction to quickly produce 3D models, which could then be examined in detail to provide a more in-depth understanding of the relevant anatomy. These models worked well when the entire procedural team wore HMDs together to plan procedures, measure the distances from the skin to sites of interest, and identify potential challenges. However, as the models were created using proprietary AI-based algorithms, it became challenging to interpret these models when particular attention to detail was needed for complex surgical hardware or complex tumors (as with Cases 1 and 4).

The manual segmentation approach with Elucis© allowed us to address these issues, as a grayscale 3D model could be customized with color-based filling and careful attention to detail. The disadvantages of this approach, however, were the significantly greater time involved in detailed model creation and the fact that model creation was dependent on the operator’s understanding of the imaging. Radiologists were not consulted when preparing models beyond the final imaging report, so conservative approximations of the size and structural involvement were utilized to avoid making a decision that could result in patient harm.

Within the field of pain management, the current literature primarily explores the role of VR pain reduction during procedures [10] and as an adjunct to traditional physical therapy and psychotherapy for chronic pain management [11]. Seong et al. [12] developed a virtual simulator for difficult injections, including superior hypogastric plexus and Gasserian ganglion blocks, allowing providers to practice procedures on custom patient models in a risk-free environment prior to the actual procedure. Others have explored the utility of augmented reality in potentially reducing procedure times and C-arm radiation exposure [13,14]. Here, we highlight how 3D models can be utilized for appropriate procedure selection and in planning an approach to performing the procedure safely. Future studies may look at the additional benefits and value created via XR pre-procedural planning, such as decreased operating or procedural times, improved efficacy of outcomes, cost-effectiveness, and patient satisfaction. Of note, we did find that certain interventional, advanced pain procedures, like PNS, which are typically performed with minimal intravenous sedation and also with minimal local anesthesia so as to allow for crucial patient feedback during the testing and placement of the electric lead, may provide certain added benefits with optimized pre-procedural planning. Improving the awake patient’s experience intraoperatively by preparing to conduct faster and more efficient surgeries is paramount to reduce the discomfort felt by patients who already suffer from chronic pain syndromes.

Some limitations to utilizing VR technology include access to hardware—although relatively low-cost headsets are becoming increasingly available—and operator skill regarding both software and translating their knowledge of anatomy. Additionally, the objective benefits of incorporating VR in terms of improving procedural times and success rates compared to traditional pre-procedural planning remain to be thoroughly investigated. Our model creation relied on pre-existing proprietary software that was not specifically coded with pain management interventions in mind. Therefore, increased time and effort was required among the model generators to create representations that were true to the patient’s anatomy and interpretable by multiple proceduralists in collaboration. Finally, we did not utilize AR for intraoperative model analysis—a next step that may further improve the purported time savings that 3D pre-procedural planning has demonstrated in other fields [15,16].

Another potential limiting factor for the use of XR in pre-procedural planning is the upfront cost and the additional time spent per patient. HMD costs range from a few hundred to a few thousand dollars, with a similar cost for the software required for model generation. Constructing models, especially using manual segmentation, can take several hours (depending on user skill and familiarity). Despite these limitations, the literature from orthopedics supports the notion that virtual models are often faster to create and utilize for planning than traditional 3D-printed models [17], and the literature from cardiology supports the use of automated segmentation in VR as an accurate and faster method of model generation compared with traditional 3D rendering [18]. Further studies may elucidate whether the upfront costs balance improvements in procedural efficacy, efficiency, and the quality of outcomes within the field of interventional pain medicine. As pain procedures are often performed on awake or lightly sedated patients, improving the time-to-procedure and intraoperative duration would be a valuable result of this technology and warrants further investigation. Nonetheless, VR provides the opportunity for in-depth and customizable procedural planning. In an academic setting, this technology can be particularly useful for the purposes of teaching and the collaborative exploration of procedural approaches.

## Data Availability

The original contributions presented in this study are included in the article. No new data were created or analyzed in this study. Data sharing is not applicable to this article.

## Abbreviations

The following abbreviations are used in this manuscript:

VR	Virtual Reality
XR	Extended Reality
AR	Augmented Reality
MR	Mixed Reality
PNS	Peripheral Nerve Stimulator
HMD	Head-Mounted Display
3D	Three-Dimensional
CT	Computerized Tomography
MRI	Magnetic Resonance Imaging
DICOM	Digital Imaging and Communications in Medicine
AI	Artificial Intelligence
2D	Two-Dimensional
NRS	Numerical Rating Scale
